# Phase I clinical trial of NH130 and the prediction of its pharmacokinetics using physiologically based pharmacokinetic modeling

**DOI:** 10.3389/fphar.2024.1474868

**Published:** 2024-09-12

**Authors:** Kun Zhang, Shanshan Zhao, Jialin Du, Lan Zhang

**Affiliations:** Phase I Clinical Trial Center, Department of Pharmacy, Xuanwu Hospital Capital Medical University, National Clinical Research Center for Geriatric Diseases, Beijing Engineering Research Center for Nerve System Drugs, Beijing Municipal Geriatric Medical Research Center, Beijing, China

**Keywords:** Parkinson’s disease, psychosis, pharmacokinetics, physiologically based pharmacokinetic model, PBPK, first-in-human

## Abstract

**Background:**

Parkinson’s disease psychosis (PDP) is a common and distressing complication of Parkinson’s disease (PD), characterized by hallucinations and delusions. This research aimed to assess the pharmacokinetics and safety of NH130, a selective serotonin 5-HT_2A_ inverse agonist, as a potential PDP treatment in healthy individuals.

**Methods:**

We conducted clinical pharmacokinetic studies and safety evaluations for NH130, employing a physiologically based pharmacokinetic (PBPK) model to predict its behavior in human body.

**Results:**

In a single-dose escalation study, healthy volunteers received NH130 at varying doses (2 mg, 6 mg, 12 mg, 24 mg, 40 mg, 60 mg, and 90 mg) or a placebo. The drug demonstrated favorable pharmacokinetics, with no serious adverse events (AEs) reported. Clinical plasma concentrations correlated well with PBPK model predictions, validating the model’s utility for guiding future clinical development.

**Conclusion:**

NH130 showed promising pharmacokinetic characteristics and safety profile, supporting its progression to multi-dose trials and suggesting its potential as a therapeutic agent for PDP.

**Clinical Trial Registration:**

http://www.chinadrugtrials.org.cn/index.html, Identifier CTR20230409.

## 1 Introduction

Parkinson’s disease (PD) is the second most prevalent neurodegenerative disease after Alzheimer’s disease, resulting primarily from damage or apoptosis of dopamine-producing cells in the brain. It typically affects individuals over the age of 60 (Camicioli et al., 2023). It is emerging as the third major cause of mortality that threatens the physical and mental health of older adults, following tumors and cardiovascular diseases (Nagy and Schrag, 2019). Approximately 10 million older individuals worldwide suffer from PD, with annual healthcare and treatment costs exceeding $57 billion, a figure that continues to increase (Aarsland and Kramberger, 2015).

PD has been considered primarily a movement disorder, with motor symptoms such as bradykinesia, myotonia, and static tremor resulting from the depletion of dopamine neurons in the substantia nigra. These symptoms constitute the main clinical manifestations of PD. However, recent evidence indicates that PD is a multi-system disease with complex clinical presentations. Parkinson’s disease psychosis (PDP) is characterized by recurrent or continuous delusions or hallucinations lasting at least 1 month. It affects up to 60% of PD patients (Forsaa et al., 2010), typically manifesting around 5–7 years before death (Kempster et al., 2010; Fenelon and Alves, 2010). Hallucinations, mainly visual, are the most common form of psychosis in PDP and may occur along with phantom smells and auditory hallucinations (Schapira et al., 2017). The emergence of psychotic symptoms often signals more aggressive disease progression and a greater need for higher levels of care, such as nursing home placement. Furthermore, PDP precedes the development of severe dementia, significantly affecting cognitive abilities, and is correlated with higher mortality rates, indicating a poorer prognosis and a faster decline in health (Peters et al., 2015; Aarsland et al., 2000).

Before April 2016, there were no effective, safe, and well-tolerated first-line drugs specifically indicated for PDP, and antipsychotic drugs without PDP indications were frequently prescribed to treat psychosis in PD patients (Reus et al., 2016). Pimavanserin was approved by the US Food and Drug Administration (FDA) in 2016 for PDP. As an inverse 5-HT_2A_ receptor agonist, it reduces the basal activity of these receptors (Garay et al., 2016; Sahli and Tarazi, 2018). Although it is the only drug approved globally for PDP, its use may be limited by factors such as cost, insurance coverage, and availability. It is not available in China. Furthermore, pimavanserin has a boxed warning about the increased risk of morbidity and mortality, as well as QT interval prolongation. Consequently, there remains a significant unmet clinical need for the development of novel, effective, and affordable drugs for PDP.

Physiologically based pharmacokinetic (PBPK) modeling is an advanced pharmacological approach that integrates knowledge of a drug’s behavior within the body (Miller et al., 2019). PBPK modeling includes the physiological and biochemical processes influencing drug absorption, distribution, metabolism, and excretion (ADME) (Parrott et al., 2013). It is a mathematical model used to fit and simulate the dynamic concentration course of drugs, serving as a vital tool in new drug development. In China, research and development efforts for PDP drugs are ongoing. NH130, a new molecular entity and highly selective serotonin 5-HT_2A_ inverse agonist, mimics pimavanserin and was recently developed by a Chinese pharmaceutical company. We report the first-in-human pharmacokinetic (PK) results and safety data from a phase I single-ascending dose study. The PBPK model was also used to simulate and predict the PK profile after oral administration of NH130.

## 2 Materials and methods

### 2.1 Pharmaceutical formulation

The active pharmaceutical ingredient (API), NH130, and its tablets are manufactured by Jiangsu Nhwa Pharmaceutical Co., Ltd. Tablets are available in 2 mg, 10 mg, and 30 mg. Placebo tablets, produced using the same formulation but without the active pharmaceutical ingredient, were also prepared.

### 2.2 Clinical trial design

This was a phase I double-blind, randomized, placebo-controlled, single ascending dose study involving healthy male and female Chinese subjects. The trial evaluated the PK, safety, and tolerability of NH130 after single oral doses. The trial was conducted exclusively at Xuanwu Hospital Capital Medical University and adhered to applicable laws, regulations, and guidelines. Ethical approval was obtained from the Ethics Committee before the initiation of the trial and the PK data modeling study (approval numbers 2022052 and Lin Yan Shen (2024) no. 009-001). The trial was registered with the China Drug Clinical Trial Registry and Information Publicity Platform (www.chinadrugtrials.org.cn) under the registry number CTR20230409. The study spanned from March 2023 to October 2023.

### 2.3 Subjects inclusion and exclusion criteria

The subjects were 18–55 years old and with a body mass index between 18.5 kg/m^2^ and 28.0 kg/m^2^. The weight criteria for participation required male subjects to weigh no less than 50.0 kg, while female subjects needed to weigh at least 45.0 kg. All subjects were required to understand the instructions, demonstrate a willingness to adhere to the study protocol and communicate effectively with the investigators.

Each exclusion criterion was designed to ensure the safety and integrity of the study results by eliminating potential confounding factors or health conditions that could affect the outcomes. Participants were excluded if they met any of the following criteria: (1) Use of prescribed or over-the-counter (OTC) medications or herbal remedies within the past 14 days; (2) Participation in other clinical trials involving investigational products in the last 3 months; (3) A history of drug or alcohol abuse within the last 2 years, or regular consumption of more than 14 standard units of alcohol per week; (4) Current smoker or cessation of smoking within the previous 12 months; (5) History of psychiatric disorders, or cardiovascular, renal, hepatic, chronic respiratory, or gastrointestinal diseases, severe adverse reactions, or serious hypersensitivity to any drug or its formulation excipients; (6) Blood donation (including components) or significant blood loss (≥200 mL) in the last 3 months; (7) Potential conflicts of interest with the study site or sponsor; and (8) Pregnancy or lactation.

### 2.4 Clinical trial protocol

Enrolled subjects underwent a 2-week screening period before admission on day −1. On this day, eligible subjects were admitted to the phase I drug clinical trial ward. On day 1, subjects were randomly assigned to receive a single oral dose of NH130 within a range of 2–90 mg or an equivalent placebo. The distribution in each group was such that ten subjects were divided, eight receiving NH130 tablets and two receiving a placebo, except in the 2 mg dose group, which comprised only two subjects, both of whom received NH130.

The experiment was conducted sequentially in a dose-escalating manner, progressing from the lowest dose group to the highest (doses: 2 mg, 6 mg, 12 mg, 24 mg, 40 mg, 60 mg, and 90 mg). Dose escalation was allowed only after the safety and tolerability of the previous group had been confirmed, maintaining a minimum interval of 24 h between adjacent dose groups. Each subject was dosed only once during the trial.

To prevent interactions with other substances, subjects were prohibited from consuming coffee, tea, soda, chocolate, alcohol, grapefruit juice, citrus juice, and any other foods or beverages containing caffeine or xanthine for 24 h before check-in and throughout their stay in the phase I clinical trial ward. We instructed subjects to fast for at least 10 h and to abstain from water for 1 hour prior to drug administration to mitigate food effects. After administration, they continued to fast for 4 h and abstained from water for 2 h. All subjects received a standard uniform meal during the trial, adhered strictly to the study protocol, and were advised to avoid strenuous physical activity. The subjects remained in-house until discharge on day 5, followed by a follow-up telephone visit on day 12, 7 days after discharge.

### 2.5 Pharmacokinetic study

The dynamics of NH130 concentration in the human body were studied by plasma sampling at predefined intervals after dose: 0 h, 0.5 h, 1.0 h, 1.5 h, 2.0 h, 3.0 h, 4.0 h, 5.0 h, 6.0 h, 8.0 h, 12.0 h, 24.0 h, 36.0 h, 48.0 h, 72.0 h, and 96.0 h (time might vary slightly between different dose groups). The determination of NH130 levels in human plasma involved a rigorous analytical procedure, starting with solid-phase extraction to purify the samples. This was followed by liquid chromatography coupled with tandem mass spectrometry (LC-MS/MS) for detection.

PK parameters were calculated using standard non-compartmental analysis with WinNonlin 6.3 software (Certara Corp., CA, United States). These parameters include the time to maximum observed plasma concentration (T_max_), maximum observed plasma concentration (C_max_), AUC from time zero to the last measurable concentration (AUC_0-t_), the area under the plasma concentration-time curve from time zero to infinity (AUC_0-∞_), apparent plasma terminal elimination half-life (T_1/2_), and apparent total plasma clearance (CL/F).

### 2.6 Dose proportionality

To check whether NH130 has linear PK properties and its dose proportionality, the PK parameters C_max_ and AUC_0-t_ were natural logarithm (ln) transformed using the power model: ln(Y) = α + β × ln (dose), where Y represents the PK parameter. The existence of a proportional relationship between the PK parameters and the doses was inferred when the 90% confidence intervals (CIs) for the slope β were contained within a specific range: $1+\frac{\ln\;\left(\theta_{L}\right)}{\ln\;\left(R\right)},1+\frac{\ln\;\left(\theta_{H}\right)}{\ln\;\left(R\right)}$ . Here, θ_L_ and θ_H_ were set at 0.8 and 1.25, respectively, signifying the lower and upper limits of dose proportionality, with R representing the ratio between the highest and lowest doses (Tung et al., 2007). Statistical analyses were conducted using SPSS 26 statistics software (IBM Corp., NY, United States). Individual C_max_, AUC_0-t_, and AUC_0-∞_ were plotted against the dose to provide an intuitive reflection of PK linearity.

### 2.7 Physiologically based pharmacokinetic model

A PBPK model was developed to simulate the PK of NH130 in humans using GastroPlus^®^ 9.9 software (Simulations Plus, Inc., CA, United States). The model comprised 14 tissue compartments representing various organs and tissues, including the lungs, adipose tissue, muscles, liver, gastrointestinal tract, spleen, heart, brain, kidneys, skin, reproductive organs, red marrow, yellow marrow, and the rest of the body. These compartments were interconnected by venous and arterial blood flow, facilitating bodily transport. Each compartment was modeled as well-stirred, assuming a uniform drug distribution within it. The model used perfusion-limited kinetics, suggesting that the drug distribution rate is predominantly governed by blood flow to each tissue.

Specific input parameters for NH130, such as molecular weight, logarithm of the partition coefficient (LogP), acid dissociation constant (pKa), and solubility, were essential to predict NH130 ADME. The PBPK software generated the predicted PK profiles and provided the corresponding PK parameters based on these inputs. Subsequent comparisons between predicted human PK data and data obtained from a single dose allowed adjustments and refinements, culminating in an optimized model to simulate the PK profile for other dosage groups. The fold error (FE), calculated as the ratio of the predicted PK parameter to the observed PK parameter, and similarly for concentrations at specific time points, served as an indicator of predictive accuracy. An FE value between 0.3 and three indicated a successful simulation (Tan et al., 2021).

### 2.8 Safety assessments

Adverse events (AEs) and changes in clinical safety-related parameters were assessed. These parameters included findings from physical examinations and essential life functions, such as body temperature, respiration rate, blood pressure, and heart rate. Clinical laboratory tests included complete blood count, serum biochemistry, urinalysis, and coagulation profiles. Assessments also included 12-lead electrocardiograms (ECGs).

## 3 Results

### 3.1 Demographics

All the volunteers were Chinese. The demographic and baseline characteristics are listed in Table 1.

**TABLE 1:** Demographic and baseline characteristics of the subjects.

	Placebo	2 mg	6 mg	12 mg	24 mg	40 mg	60 mg	90 mg
N	12	2	8	8	8	8	8	8
Age (years)	30.2 (5.42)	33.5 (19.09)	32.9 (8.75)	33.6 (8.88)	31.7 (8.43)	37.5 (9.40)	35.6 (7.97)	38.4 (9.69)
Sex								
Male Female	6 (50.0) 6 (50.0)	1 (50.0) 1 (50.0)	4 (50.0) 4 (50.0)	4 (50.0) 4 (50.0)	4 (50.0) 4 (50.0)	4 (50.0) 4 (50.0)	4 (50.0) 4 (50.0)	3 (37.5) 5 (62.5)
Height (cm)	166.15 (11.506)	166.75 (13.789)	167.39 (6.754)	166.00 (8.000)	162.89 (9.499)	163.56 (8.029)	163.61 (6.397)	164.25 (6.803)
Weight (kg)	60.94 (10.025)	62.15 (2.333)	63.74 (7.917)	62.91 (7.941)	63.61 (8.126)	62.31 (5.197)	61.83 (5.151)	60.58 (6.699)
BMI (kg/m^2^)	22.05 (1.707)	22.60 (2.687)	22.71 (2.109)	22.84 (2.052)	23.91 (1.719)	23.36 (1.699)	23.11 (2.147)	22.60 (2.467)

Mean (SD) values are presented above, except for sex, in which the numbers in the parenthesis mean percent.

### 3.2 Pharmacokinetic study

**FIGURE 1:**
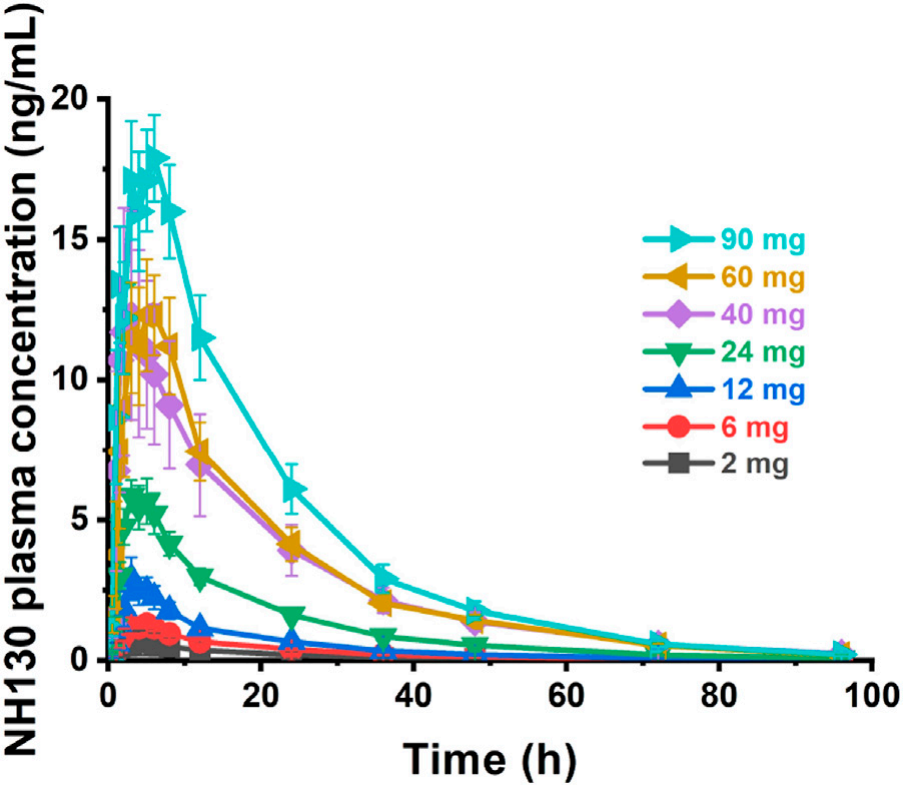
NH130 plasma concentrations following a single oral dose to healthy subjects. The presented data are group mean results ± SEM. N = 2 for the 2 mg dose group, and N = 8 for other dose groups.

Plasma concentration profiles plotted against time are shown in Figure 1. The individual NH130 concentrations were used to compute the PK parameters (Table 2). Exposure exhibited an apparent dose-proportional increase. AUC_0-t_ are close to AUC_0-∞_. However, there was considerable inter-subject variability (geometric coefficient of variation, CV%) associated with exposure, particularly for C_max_, AUC_0-t_, and AUC_0-∞_ across the dose range. The T_1/2_ of NH130 was approximately 15.7 h (range: 13.7–18.2 h) and was independent of dose. Clearance rates ranged from 168 L/h to 304 L/h. The lowest T_1/2_ and CL/F occurred in the 2 mg dose group.

**TABLE 2:** NH130 pharmacokinetic parameters.

	2 mg	6 mg	12 mg	24 mg	40 mg	60 mg	90 mg
T_max_ (h)	3.50 (2.00, 5.00)	4.00 (3.00, 6.00)	4.00 (3.00, 6.00)	3.00 (2.00, 5.00)	4.50 (1.50, 6.00)	3.50 (1.50, 6.12)	3.00 (1.00, 8.00)
C_max_ (ng/mL)	0.732 (86.2%)	1.43 (36.7%)	2.50 (76.5%)	6.51 (31.4%)	11.4 (82.6%)	13.5 (47.1%)	20.5 (18.1%)
AUC_0-t_ (h×ng/mL)	10.8 (61.2%)	23.0 (26.5%)	37.7 (66.5%)	105 (28.5%)	215 (68.7%)	253 (45.4%)	364 (35.1%)
AUC_0-∞_ (h×ng/mL)	11.9 (59.1%)	24.7 (24.7%)	39.4 (62.9%)	107 (27.8%)	220 (68.6%)	257 (45.1%)	369 (35.5%)
T_1/2_ (h)	13.7 (2.7%)	15.7 (15.5%)	14.6 (17.0%)	15.5 (15.7%)	18.2 (9.8%)	16.4 (6.9%)	14.9 (13.7%)
CL/F (L/h)	168 (59.1%)	243 (24.7%)	304 (62.9%)	224 (27.8%)	182 (68.6%)	234 (45.1%)	234 (35.5%)

Geometric mean (geometric CV%) values are presented above except T_max_, where the median (range) is presented.

**FIGURE 2:**
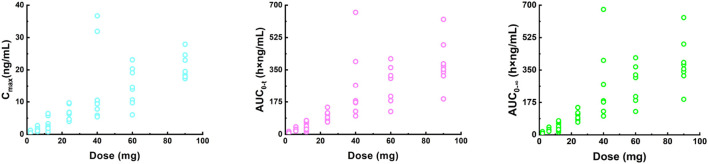
Plot of individual C_max_ (left), AUC_0-t_ (middle) and AUC_0-∞_ (right) values versus dose in a single ascending dose study (2, 6, 12, 24, 40, 60, and 90 mg). C_max_, the maximum plasma concentration; AUC_0-t_, the area under the plasma concentration-time curve from 0 to the last measurement time; AUC_0-∞_, the area under the plasma concentration-time curve from time zero to infinity.

The C_max_, AUC_0-t_, and AUC_0-∞_ values increased approximately 28-fold (from 0.732 ng/mL to 20.5 ng/mL), 33.7-fold (from 10.8 h×ng/mL to 364 h×ng/mL), and 31-fold (from 11.9 h×ng/mL to 369 h×ng/mL) with a 45-fold increase in doses (from 2 mg to 90 mg), respectively. The three parameters from all the individuals were plotted separately for the dose, we can see that there is an apparent gradual upward linear trend, although some dose groups showed significant deviation (Figure 2). The data points for the 40 mg group were more scattered than those of other dose groups. A power model was used to evaluate dose proportionality (Table 3). The β values for C_max_, AUC_0-t_, and AUC_0-∞_ fell within the predefined acceptance range. However, the 90% CIs for β of C_max_, AUC_0-t_, and AUC_0-∞_ were larger than and covered the acceptance range. All the values of the three β were close to 1.

**TABLE 3:** Assessment of dose proportionality of NH130 based on the power model.

Pharmacokinetics parameters	β	90% confidence interval	Acceptance range	Dose range (mg)
C_max_	0.97	(0.86, 1.08)	0.94–1.06	2 mg-90
AUC_0-t_	1.04	(0.93, 1.14)		
AUC_0-∞_	1.02	(0.91, 1.12)		

C_max_, maximum plasma concentration; AUC_0-t_, the area under the plasma concentration-time curve from 0 to the last measurement time. AUC_0-∞_, the area under the plasma concentration-time curve from time zero to infinity.

### 3.3 Physiologically based pharmacokinetic model

The physicochemical and ADME properties of NH130 were measured in *in vitro* experiments or predicted and optimized using GastroPlus^®^ software. For some missing data or information not easily obtained, default parameters or system built-in modes were used. To establish the PBPK model, the tissue-to-plasma partition coefficient (Kp) values were simultaneously optimized based on the system-built-in Lukacova (Rodgers-Single) method. The PBPK model was optimized and validated using observed PK data obtained after a single dose. The PK data of the 60 mg dose group was used as a reference to benchmark to fit the PBPK model, while other dose groups were used to test and verify the model. The main input parameters are shown in Table 4.

**TABLE 4:** The input parameters for NH130 in GastroPlus software.

Parameters	Value	Source
Molecular weight	423.52 g/mol	Calculated value
LogP	4.1	Data from Jiangsu Nhwa Pharmaceutical Co., Ltd.
pKa	1.949, 5.2, 6.1	Same as above
Solubility	5.87 mg/mL	Same as above
Apparent permeability coefficient	0.8 × 10^−6^ cm/s	Optimized value
Kp Lung Adipose Muscle Liver Spleen Heart Brain Kidney Skin Reproductive organ Red marrow Yellow marrow Rest of body	0.51 19.88 1.75 2.85 1.78 1.20 4.50 1.78 2.29 1.79 5.00 19.88 1.80	Same as above

LogP, logarithm of the partition coefficient; pKa, acid dissociation constant; Kp, tissue-to-plasma partition coefficient.

**FIGURE 3:**
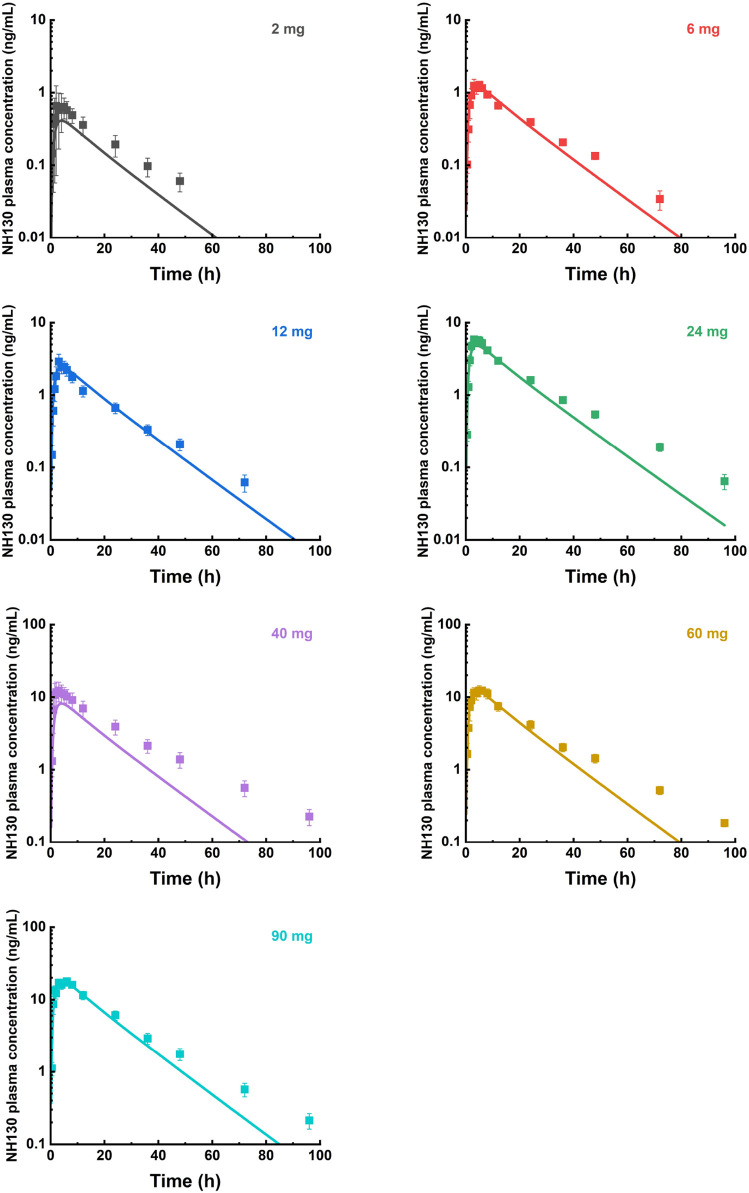
Mean observed (symbol ± SEM) and simulated (lines) plasma concentrations for 2 mg, 6 mg, 12 mg, 24 mg, 40 mg, 60 mg, and 90 mg single doses with the physiologically based pharmacokinetic model. N = 2 for the 2 mg dose group, and N = 8 for other dose groups.

The predicted and observed plasma concentration-time profiles before 40 h were superimposed for the 6 mg, 12 mg, 24 mg, 60 mg, and 90 mg dose groups (Figure 3). Although the trend is consistent, the predicted PK curves did not align well with the observed data for the 2 mg and 40 mg dose groups. The elimination phases of all dose groups after 40 h were not well-fitted. The accuracy of the PBPK model was evaluated by FE. The predicted and observed C_max_ and AUC_0-∞_, as well as FE for all dose groups, are listed in Table 5, with all FE of PK parameters within a 2-fold range.

**TABLE 5:** The predicted and observed values of C_max_ and AUC_0-∞_ after each dose administration of NH130 to human.

Dose (mg)	C_max_ (ng/mL)			AUC_0-∞_ (h×ng/mL)		
	Predicted	Observed	Fold error	Predicted	Observed	Fold error
2	0.41	0.66	0.62	7.62	13.37	0.57
6	1.23	1.28	0.96	22.86	25.33	0.90
12	2.45	2.90	0.84	45.72	45.30	1.01
24	4.85	5.84	0.80	91.42	112.84	0.81
40	8.15	12.30	0.66	152.45	270.40	0.56
60	12.26	12.30	1.00	228.63	278.36	0.82
90	18.54	17.90	1.04	343.01	398.77	0.86

**FIGURE 4:**
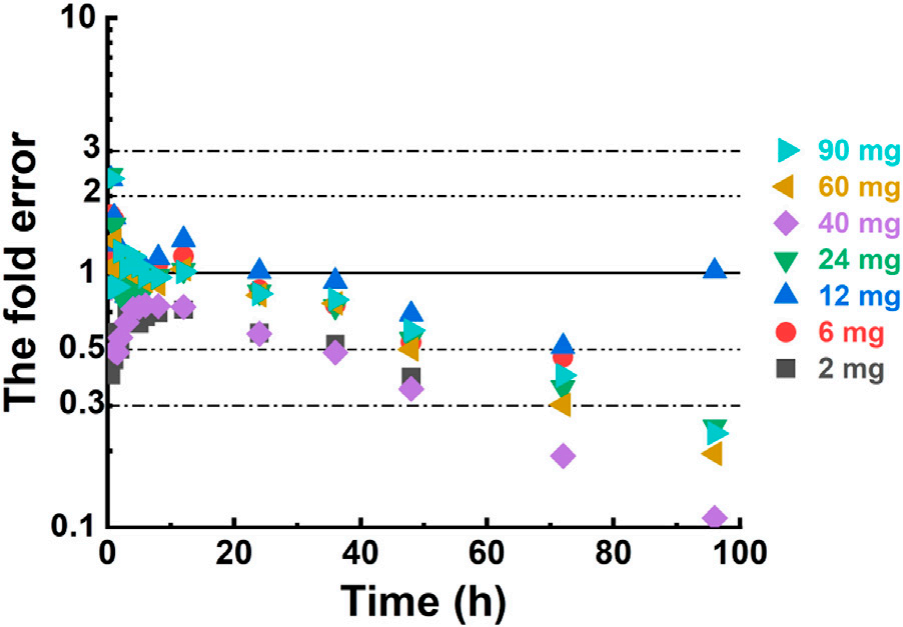
The fold error of all the predicted and observed concentration data points.

For individual concentration data points, most FE values were within a 2-fold range before 40 h (Figure 4), implying a successful prediction. However, there were outliers. For example, the FE value of the data points at 0.5 h in the 12 mg, 24 mg, and 90 mg dose groups was within 3 times but beyond 2 times. Most of the FE values at 96 h were greater than 3 times, indicating a deviation of the predicted data points from the observed data points at that time.

### 3.4 Safety findings

The overall incidence of AEs was low (Table 6). All AEs were mild or moderate in severity. There were no deaths or severe adverse events (SAEs). As the dose increased gradually, the incidence of treatment-emergent adverse events (TEAEs) also increased. The most common TEAEs were neurological symptoms, such as dizziness or drowsiness, gastrointestinal symptoms, such as nausea or vomiting, and QT interval prolongation (Table 7). Women predominantly experienced orthostatic hypotension or postural low blood pressure, classified as a moderate AE. No significant clinical impacts were detected on vital signs, body weight, blood glucose levels, or lipid profiles.

**TABLE 6:** Incidence of adverse events.

	Placebo	2 mg	6 mg	12 mg	24 mg	40 mg	60 mg	90 mg
Adverse events (AEs)	4 (33.3)	2 (100.0)	0	2 (25.0)	3 (37.5)	3 (37.5)	5 (62.5)	5 (62.5)
Treatment-emergent adverse events (TEAEs)	4 (33.3)	2 (100.0)	0	2 (25.0)	3 (37.5)	3 (37.5)	5 (62.5)	5 (62.5)
Investigational medicinal product-related TEAEs	2 (16.7)	0	0	0	2 (25.0)	2 (25.0)	3 (37.5)	4 (50.0)
Serious TEAEs	0	0	0	0	0	0	0	0

The numbers in the table represent the number of subjects. The numbers in parenthesis mean percent.

**TABLE 7:** Classification of investigational medicinal product-related treatment-emergent adverse events.

	Placebo	2 mg	6 mg	12 mg	24 mg	40 mg	60 mg	90 mg
Various inspection								
ECG QT interval prolongation	0	0	0	0	2	0	1	1
Decreased white blood cell count	1	0	0	0	0	0	0	0
ECG PR shortening	1	0	0	0	0	0	0	0
Elevated heart rate	0	0	0	0	0	1	0	0
Neurological diseases								
Giddy	0	0	0	0	0	0	3	2
Drowsy	0	0	0	0	0	1	2	1
Headache	0	0	0	0	0	0	0	1
Presyncope	0	0	0	0	0	0	1	0
Gastrointestinal diseases								
Nausea	0	0	0	0	0	0	2	2
Vomiting	0	0	0	0	0	0	1	1
Diarrhea	0	0	0	0	0	0	0	1
Vascular and lymphatic diseases								
Orthostatic hypotension	0	0	0	0	0	0	2	1
Heart organ disease								
Intra-chamber conduction disturbance	0	0	0	0	0	1	0	0
First-degree atrioventricular block	0	0	0	0	0	0	0	1

The numbers in the table represent the number of cases. More than one adverse event may occur to a subject.

## 4 Discussion

Many drugs have been used to treat PDP, driven by emerging preclinical and clinical research. For example, recently, novel antipsychotics such as saracatinib, a Src kinase inhibitor, and SEP-363856, a 5-HT_1A_ and trace amine-associated receptor 1 (TAAR1) agonist, have received attention (Dedic et al., 2019). Several early-stage clinical trials are underway (Nih, 2018; Nih, 2016), while others are in the planning phase (Davies and Bhattacharyya, 2019; Blair et al., 2015). However, some clinical trial results have been disappointing, with limited evidence supporting their efficacy in preventing PDP and the potential to worsen motor function (Leroi et al., 2004).

This first-in-human clinical study investigated the PK, safety, and tolerability of single ascending doses of NH130 in healthy male and female Chinese subjects. The PK profile of NH130 was characterized in these subjects, ranging from 2 mg to 90 mg in a single dose. The NH130 tablet demonstrated rapid absorption and linear PK properties. Its maximal plasma concentrations were achieved three–4.5 h after oral administration, with a half-life of approximately 13.7–18.2 h. NH130 demonstrated good tolerability with no reports of SAEs. TEAEs were mostly mild or moderate, and no dose-limiting AEs were observed.

A drug with linear PK properties is highly desirable for clinical use because it simplifies dose adjustment and dosing schedules. A key aspect of linear PK is dose proportionality. During the clinical development of a new drug, assessing dose proportionality is a fundamental PK analysis that involves administering various doses and analyzing the resulting concentration-time data to confirm the linear relationship.

As an exploratory-stage drug clinical trial, there were seven dose groups in this study, ranging from 2 mg to 90 mg. The wide dose range resulted in a high R-value in the dose proportionality calculation step, leading to a narrow acceptance range within the 90% CI of β for C_max_, AUC_0-t_, and AUC_0-∞_. This phase I clinical trial laid the foundation for future studies. A planned multi-dose escalating clinical trial with such a wide dose range will not be needed. The results suggested that the primary PK parameters, C_max_, AUC_0-t_, and AUC_0-∞_ of NH130 have linear kinetic characteristics after a single dose within this dose range.

Participants were initially in good health and were instructed to abstain from taking concomitant medications, supplements, and foods that could potentially induce or inhibit drug transporters or enzymes. They were required to avoid taking such substances throughout the study. These measures were implemented to minimize inter-individual differences in the absorption or clearance of NH130. Despite these precautions, there was still high PK variability in some dose groups, such as the 40 mg group. Several factors may contribute to this variability. At this stage, this can only be attributed to the heterogeneity of the subjects.

The integration of mechanistic approaches with *in vitro* data is gaining prominence in the pharmaceutical industry, particularly for forecasting the oral absorption of novel chemical entities. Such foresight is vital for crafting effective clinical trial frameworks and formulating robust drug development strategies. A comprehensive grasp of a drug’s physicochemical characteristics and its influence on oral absorption is fundamental to any modeling and simulation endeavor. By providing precise forecasts, these models can significantly minimize compound attrition rates throughout drug development, leading to substantial savings in cost and time related to clinical trials. The concentration of the drug distributed in each tissue is dynamically balanced, with the Kp values being modified based on the commonly applied system built-in Lukacova (Rodgers-Single) method. This method is widely used in PBPK modeling to estimate drug distribution across tissues. It has been validated and applied to various compounds and scenarios, demonstrating its robustness and versatility. PBPK modeling and simulation were used to fit and simulate the PK of NH130 and to predict the PK profile with increasing doses after a single dose. This is also the first study to report a PBPK model of NH130. Pre-clinical studies showed that although the primary route of NH130 elimination is metabolic, with excretion occurring in metabolites, these metabolites have no therapeutic activity and negligible toxic effects. Consequently, the metabolites were not considered. Possible enzymes or transporters involved were also excluded to avoid creating an overly complex model. The predicted and observed data in the elimination phase for all dose groups did not fit well after 40 h of the dose. However, this is not a significant concern because NH130 is designed to be administered to patients at least once daily. Therefore, a preferable fitting within 24 h after dosing is sufficient. The deviation may be because a tissue explicitly binds a small amount of the drug and continuously and slowly releases it, resulting in the blood drug concentration being maintained at a low level for a longer time. The current model did not consider this mechanism, leading to an underestimation of the prediction of the terminal elimination phase.

The deviation of predicted data from observed data in the 2 mg and 40 mg dose groups may be caused by the small sample size and inter-subject variation. For the 2 mg dose group, there was only one female and one male subject. For the 40 mg dose group, C_max_, AUC_0-t_, and AUC_0-∞_ of several subjects were significantly higher than the others, even exceeding those of the 90 mg dose group. One potential consideration for the inter-subject variation could be ascribed to gene polymorphism. Unfortunately, there was no research result about genes in the pre-clinical study at present time. Similarly, there are no statements about pharmacogenomics in pimavanserin instruction. Additional data from pre-clinical studies and subsequent clinical trials are needed to clarify this abnormal variation. Several FE values at 0.5 and 96 h were outside the 2-fold or 3-fold range. Errors in blood sampling or detection, particularly at the first and last time points, may cause data discrepancies. Despite these potential errors, the overall fit of the data remains adequate, indicating that the PBPK models in humans are accurate and reliable. This suggests that the models are sufficiently robust and can be trusted to predict NH130 drug behavior in clinical settings. As our understanding of NH130 continues to evolve through ongoing pre-clinical research, and should the need arise for more nuanced predictions, such as pharmacodynamics (PDs) assessments, we will revisit the model. At that juncture, we will consider incorporating other relevant factors into the PBPK model to enhance its predictive capabilities and align with our advanced research requirements.

The AEs observed were generally mild to moderate. The clinical trial protocol, which required fasting for more than 10 h before drug administration and involved intensive blood sampling, was identified as a significant contributor to the incidence of postural hypotension. In addition to the possible drug-related side effects, factors such as weak autonomic nervous system regulation in some female subjects contributed to these problems.

From published clinical trials we know that pimavanserin has the potential to cause some neurological and gastrointestinal symptoms. Like other antipsychotics, pimavanserin may also cause QT interval prolongation, which could potentially lead to *torsades de pointes*. Similar to pimavanserin, NH130 also has neurological and gastrointestinal side effects, as well as the adverse reaction of QT interval prolongation. The small sample size provided limited evidence for the incidence rate of NH130. These types of AE should be emphasized and closely monitored in future multi-dose and long-term efficacy and safety studies. In future studies, the sample size for NH130 clinical trials will be expanded, the PBPK model will be optimized further based on more pre-clinical and clinical study results, and the in-depth mechanism of NH130 will be elucidated. Additionally, PK/PD studies can be introduced and bridged to facilitate the study of the effect of NH130.

## 5 Conclusion

NH130, a highly selective serotonin 5-HT_2A_ inverse agonist, demonstrated good safety and tolerability in a single ascending dose phase I study in healthy Chinese subjects. The results are the foundation for further evaluation of multi-dose PK and safety studies. Considering the advantages and limitations of the study, the PBPK model will be further optimized based on more results from future pre-clinical studies and expanded clinical trials. Clinical trials of NH130 in patients with PDP should also be considered and prioritized.

## Data Availability

The raw data supporting the conclusions of this article will be made available by the corresponding author upon reasonable request.
